# FTO-mediated autophagy promotes progression of clear cell renal cell carcinoma via regulating SIK2 mRNA stability

**DOI:** 10.7150/ijbs.77774

**Published:** 2022-10-03

**Authors:** Yawei Xu, Jingcheng Zhou, Lei Li, Wuping Yang, Zedan Zhang, Kenan Zhang, Kaifang Ma, Haibiao Xie, Zheng Zhang, Lin Cai, Yanqing Gong, Kan Gong

**Affiliations:** 1 Department of Urology, Peking University First Hospital, Institute of Urology, Peking University, National Urological Cancer Center, Beijing Key Laboratory of Urogenital Diseases (Male) Molecular Diagnosis and Treatment Center, Beijing, China.; 2 Department of Urology, Guangdong Provincial People's Hospital, Guangdong Academy of Medical Sciences, Guangzhou, China.

**Keywords:** FTO, autophagy, SIK2, ccRCC

## Abstract

The progression of clear cell renal cell carcinoma (ccRCC) remains a major challenge in clinical practice, and elucidation of the molecular drivers of malignancy progression is critical for the development of effective therapeutic targets. Recent studies have demonstrated that N^6^-methyladenosine (m^6^A) is the most abundant modification of eukaryotic mRNA and plays a key role in tumorigenesis and progression. However, the biological roles and underlying mechanisms of m^6^A-mediated autophagy in cancers especially in ccRCC remain poorly elucidated. m^6^A dot blot assay, m^6^A RNA methylation assay kit and immunofluorescence analysis were used to profile m^6^A levels in tissue samples and their correlation with autophagic flux. Expression patterns and clinical significance of fat mass and obesity-associated protein (FTO) were determined through bioinformatics analysis, real-time PCR, western blotting, immunohistochemistry. RNA-seq, MeRIP-seq, MeRIP-qRT-PCR, RIP-qRT-PCR, transmission electron microscopy, immunofluorescence analysis and luciferase reporter assay were used to investigate the underlying mechanism of the FTO-autophagy axis. The role of FTO and autophagy in ccRCC progression was evaluated both *in vitro* and *in vivo*. Here we found that m^6^A modification was suppressed and closely related to autophagic flux in ccRCC. Elevated FTO was inhibited by rapamycin, whereas silencing FTO enhanced autophagic flux and impaired ccRCC growth and metastasis. SIK2 was identified as a functional target of m^6^A-mediated autophagy, thereby prompting FTO to play a conserved and important role in inhibiting autophagy and promoting tumorigenesis through an m^6^A-IGF2BP2 dependent mechanism. Moreover, the small molecule inhibitor FB23-2 targeting FTO inhibited tumor growth and prolonged survival in the patient-derived xenograft (PDX) model mice, suggesting that FTO is a potential effective therapeutic target for ccRCC. Our findings uncovered the crucial role of FTO/autophagy/SIK2 axis in modulating the progression of ccRCC, suggesting that FTO may serve as a valuable prognostic biomarker and promising therapeutic target in ccRCC.

## Introduction

Renal cell carcinoma (RCC) is among the ten most common malignancies in both men and women worldwide, accounting for 4.2% of all new cancer cases 1. The most common histological subtype clear cell RCC (ccRCC) derives from the epithelial cells of the proximal renal tubule and accounts for most of the cancer-related deaths 2. It is estimated that over 30% of ccRCC patients present with distant metastasis at diagnosis. Targeted therapy and immunotherapy improve overall survival, but interindividual variability in treatment response and the resistance that most patients eventually develop leads to a 5-year survival rate of only 8% in patients with metastatic ccRCC 3. Elucidating the molecular drivers of ccRCC progression and metastasis is critical for the development of effective treatments for advanced ccRCC.

Cancer cells acquire the ability of progression and distant metastasis through genetic and epigenetic changes, one of which is the modification of mRNA transcripts 4. m^6^A modification affect mRNA stability, subcellular localization, alternative splicing, RNA-protein interactions, transport, translation, and transcription 5. m^6^A modification play critical roles in the directed differentiation of haematopoietic stem cells, the maintenance of embryonic stem cell pluripotency, the regulation of spermatogenesis, and diseases such as cancer 6. N^6^-methyladenosine (m^6^A) resulting from methylation modification is the most abundant form of internal RNA modification in eukaryotes. In mammalian cells, m^6^A modification is dynamic and reversible. It is catalyzed by m^6^A methyltransferases (METTL3, METEL14, and WTAP), also known as “writers”. m^6^A demethylases (FTO and ALKBH5), also known as “erasers”, can directly remove m^6^A modifications from mRNA. In addition, specific RNA-binding proteins, also known as “readers”, are responsible for specifically recognizing and binding methylated sites, thereby exerting specific biological functions 7. FTO (fat mass and obesity-associated protein) is a gene involved in the regulation of adipogenesis and energy intake 8, and its common variants are associated with body mass index and obesity from childhood to the old age 9. FTO was the first m^6^A demethylase identified among these m^6^A effectors and has been shown to play oncogenic roles in non-small cell lung cancer 10, esophageal squamous cell carcinoma 11, gastric cancer 12, and leukemia 13. However, the role of RNA methylation in the tumorigenesis and progression of ccRCC remains unclear.

Autophagy is an evolutionarily conserved catabolic degradation mechanism within cells in which cytoplasmic materials, proteins, damaged organelles and lipids are sequestered into vesicles called autophagosomes for degradation and recycling 14. The mechanism by which FTO regulates autophagy remains unclear. Recently, a study showed that knockdown of FTO resulted in attenuated autophagosome formation, thereby inhibiting autophagy and adipogenesis 8. Conversely, another study found that silencing FTO in oral squamous cell carcinoma cells increased autophagic flux and suppressed tumorigenesis 15. However, the mechanism by which FTO-mediated autophagy regulates ccRCC tumorigenesis and progression is still poorly understood.

In this study, we discovered that FTO was significantly upregulated in ccRCC, and silencing FTO expression increased autophagic flux. Our study reveals that FTO reduces SIK2 mRNA stability through an m6A-IGF2BP2-dependent pathway, leading to ccRCC proliferation and metastasis. Taken together, our study suggests a new potential prognostic biomarker and therapeutic target for patients with ccRCC.

## Materials and Methods

### Patients, specimens and cell culture

This study was based on guidelines of the Declaration of Helsinki and approved by the Institutional Ethical Board of Peking University First Hospital (Beijing, China, BMU2018JI002). All tissue samples were obtained from patients undergoing renal tumor resection at Peking University First Hospital and independently diagnosed as ccRCC by two senior urological pathologists. Between April 2012 to August 2012, 99 pairs of adjacent non-tumor and ccRCC tissue samples were collected for tissue microarray analysis. For ccRCC-1 cohort, tumor tissues and adjacent non-tumor tissues from 99 ccRCC patients who underwent resection between April 2012 to August 2012 were collected to the construction of tissue microarray (TMA). This cohort was used to assess the expression levels and clinical significance of FTO. For ccRCC-2 cohort, RNA was extracted from 24 pairs of frozen adjacent non-tumor and ccRCC tissues for quantitative real-time PCR (qPCR) assay and total RNA m^6^A modification level quantification.

The human ccRCC cell lines (OSRC-2, 786-O, Caki-1, 769-P, ACHN, and A498) and the epithelial cell line of proximal convoluted tubules in human renal cortex (HK2) were purchase from the American Type Culture Collection (ATCC, Manassas, VA, USA). OSRC-2, 786-O, and 769P cells were cultured in RPMI-1640 (Invitrogen-Gibico), and Caki-1, ACHN, A498, and HK2 cells were cultured in DMEM (Gibico). All cells culture media contained 10% fetal bovine serum (Thermo Fisher Scientific) and 1% penicillin-streptomycin (Gibco). All cells were grown at 37℃ in a standard humidified incubator with 5% CO2 and 95% O2. For function analysis, 10 mM 3MA or 10 μM chloroquine (MedChemExpress) was added to the medium to inhibit autophagy, while 100 nM rapamycin (MedChemExpress) was used to induce autophagy. For the actinomycin D assay, 2 μg/ml actinomycin D (Sigma-Aldrich) was added to OSRC-2 and 786-O cells and the cells were cultured for 0, 6, 12, and 18 hours.

### RNA extraction and qRT-PCR

Total RNA was extracted from ccRCC cells and frozen tissues using TRIzol reagent (Invitrogen) and the cDNA was generated by M-MLV reverse transcriptase (Invitrogen) following the manufacturer's instructions. qRT-PCR analysis was performed using SYBR Green PCR Master Mix (Roche) with the Agilent Technologies AriaMx Real-Time PCR System (Agilent). GAPDH served as a normalized internal control and the relative expression of each gene was analyzed using the 2- ∆∆Ct method. All primers used in this study are listed in Table S1.

### Total RNA m^6^A quantification

Total m^6^A levels in mRNA were detected using the m^6^A RNA methylation assay kit (Abcam) according to the manufacturer's protocol. Briefly, 200 ng of RNA was added to assay wells, followed by the appropriate dilution of detection antibody to each well. The absorbance value at 450 nm was detected by a Varioskan Flash microplate reader (Thermo Scientific, USA), and the relative level of m^6^A RNA methylation in total RNA was calculated according to the following formula: m^6^A% = [(Sample OD-NC OD) ÷S]/ [(PC OD-NC OD) ÷P] ×100% (S is the amount of input sample RNA in ng, P is the amount of input Positive Control in ng).

### m^6^A dot blot assay

The dot blot assay was performed according to the bio-protocol database (https://en.bio-protocol.org/e2095). Briefly, RNA samples were spotted onto Amersham Hybond-N+ membranes (GE Healthcare) installed in a Bio-Dot Apparatus (Bio-Rad) followed by UV cross-linking. Stained with 0.02% methylene blue (Solarbio, China) and scanned to indicate the total input RNA content. After drying, the membrane was blocked with 5% BSA for 1 hour and incubated with specific anti-m^6^A antibody (Synaptic Systems, 1:1000) overnight at 4 °C with gentle shaking. Subsequently, the membrane was incubated with HRP-conjugated anti-mouse immunoglobulin G (Abcam, 1:3000) for 1 hour at room temperature. Finally, the membranes were detected with a Chemiluminescent HRP substrate kit (Millipore, USA) and the images were acquired.

### Western blot analysis and antibodies

Protein extracts from tissues or cells were obtained by pre-cooled RIPA buffer (Beyotime, Shanghai, China) containing proteases and phosphatases inhibitors (Beyotime, Shanghai, China). After quantification of protein concentration by BCA assay kit (Pierce), samples were heated at 90 °C for 10 min. Protein abundance was detected and analyzed by SDS-polyacrylamide gel electrophoresis and immunoblotting as described in previous studies 16. Primary antibodies used in this study were listed in Table S2.

### Immunofluorescence analysis

Cells were first washed with phosphate buffered saline (PBS) and fixed with 4% paraformaldehyde for 10 min at room temperature. Permeabilization with Triton X-100 (Beyotime Biotechnology, P0096) for 10 minutes followed by blocking with immunostaining blocking buffer for 1 hour. Subsequently, Cells were incubated with primary antibody overnight at 4 °C, followed by incubation with secondary antibodies. The images were taken by a FV3000 confocal laser microscope (Olympus, Tokyo, Japan). LC3 puncta between 0.3 μM and 1.0 μM in diameter were defined as positive puncta as described in previous studies 8.

### Immunohistochemistry (IHC) staining

Tissue samples were fixed in 4% paraformaldehyde overnight and embedded in paraffin. Tissue sections were stained with hematoxylin and eosin (H&E) for histological analysis. The IHC score was calculated by multiplying the staining intensity of cells that stained positively (no staining = 0, weak staining = 1, moderate staining = 2, and strong intense staining = 3) by the score for the percentage of positive cells (no positive staining = 0, 1%-25% = 1, 26%-50% = 2, 51%-100% = 3), resulting in a score range of 0-9. All scoring was performed independently by two senior urologic pathologists.

### Plasmids and transfection

Three different lentiviral shRNA oligonucleotide sequences for knockdown of FTO and IGF2BP2, respectively, were synthesized from Syngentech (Beijing, China). Gene sequences coding for FTO, SIK2-WT, and SIK2-MUTs were cloned into the pcDNA3.1 (Invitrogen) vector according to the manufacturer's instructions. The most validated sequences were used for subsequent assays. The transfection of plasmids was performed using Lipofectamine 3000 according to the manufacturer's protocols.

### Transmission electron microscopy (TEM)

TEM analysis was performed as described in previous studies 8. Briefly, cells to be assayed were washed twice with PBS and fixed in 1.5% glutaraldehyde for 4 hours, followed by post-fixed 1% osmium tetroxide for 2 hours. Double-distilled water was used to wash the samples for 5 minutes each time. Samples were dehydrated in increasing concentrations of ethanol (50%, 70%, 90% and 100%) and anhydrous acetone (3 times, 15 minutes each time). Saturate the samples sequentially with 1:1 and 1:2 mixtures of acetone and embedding medium. The samples were transferred to a 37 °C oven for 12 hours, followed by 48 hours at 60 °C to complete the polymerization procedure. 60 nm ultrathin sections (LEICA EM UC7) stained with 1% uranyl acetate and imaged using a JEM-1400Flash (JEOL, Japan) transmission electron microscope.

### Cell proliferation assay

The transfected cells were seeded in 96-well plates at a density of 2000 cells per well with fresh medium. The cell proliferation was detected using CCK8 kit (Dojindo Laboratories, Kumamoto, Japan) at time in 0, 24, 48, 72, and 96 h. The cells were incubated with 10 μl CCK8 reagent at 37 °C for 1h. The absorbance was measured at 450 nm using a Varioskan Flash microplate reader (Thermo Scientific, USA).

### Colony formation assay

Seed 500 cells per well in 6-well plates and culture in an incubator for 14 days. Colonies were fixed in 4% paraformaldehyde for 20 minutes and subsequently stained with 0.1% crystal violet (Beyotime, China). Stained colonies were then imaged and manually counted.

### Cell migration and invasion assays

In transwell migration and invasion assays, starved ccRCC cells were seeded into the upper chamber (Corning, USA) with (transwell invasion assay) or without (transwell migration assay) Matrigel (BD Biosciences, USA). Medium without fetal bovine serum and ccRCC cells (1-5×10^4^ cells/200 μl) were added to the upper chamber, and 10% fetal bovine serum was added to the lower chamber medium as the attractant. Cells on the upper surface of the chamber were removed after 24 hours of culture, and cells on the lower surface were fixed with 4% paraformaldehyde and stained in 0.1% crystal violet. Finally, count the stained cells under the microscope.

### RNA immunoprecipitation (RIP)-qRT-RCR

RIP assays were performed using Magna RIP Quad RNA-Binding Protein Immunoprecipitation Kit (Merck, USA) according to the manufacturer's protocols. Briefly, cells were lysed with cell lysate containing protease and RNase inhibitors, and cell supernatants were incubated with anti-IGF2BP2 antibody-conjugated beads overnight at 4 °C. The beads were washed with buffer and treated with proteinase K (Beyotime, China) for 1 hour. Then, total RNA was extracted from the supernatant using TRIzol reagent for qRT-PCR analysis.

### MeRIP-seq and MeRIP-qPCR

Total RNA was first extracted from OSRC-2 cells treated with or without 100nM rapamycin for 72h. Fragmented RNA was purified, approximately 1/10 of which was saved as input control, and the remainder was incubated with anti-m^6^A antibody for 2 hours, followed by incubation with ore-washed Protein A/G Magnetic Beads in immunoprecipitation buffer at 4 °C overnight. Subsequently, library preparation and MeRIP-seq was performed by OE Biotech (Shanghai, China) as described in previous studies 17. For MeRIP-qPCR, a portion of the precipitated product was reverse transcribed by PCR and analyzed.

### mRNA stability assays

mRNA stability analysis was performed as described in previous studies 8, 18. In brief, 5 μg/ml of Actinomycin D (Merck, USA) was used to block cellular mRNA transcription. Cells were harvested after incubation for the indicated times, and total RNA was extracted for reverse transcription. The mRNA transcription level of target gene was detected by qPCR, and the half-life of mRNA was calculated.

### Luciferase reporter assay

ccRCC cells were seeded in 6-well plates and cultured to about 70% confluence, then the cells were transfected with plasmids for SIK2-WT or SIK2-MUTs. After 48 hours, measure the luciferase intensity according to the manufacturer's instructions. Luciferase activity was detected by a Dual Luciferase Reporter Gene Assay Kit (KeyGEN BioTECH, China). The Firefly luciferase was used to normalize the activity of Renilla luciferase and to evaluate the expression efficiency.

### Orthotopic tumor growth

Animal studies in this study was approved by the Laboratory Animal Ethics Committee of Peking University First Hospital (J2022008, Beijing, China), and all details abide by the ethical principles of laboratory animal welfare and was supervised by the committee and laboratory managers. 6-week-old B-NDG mice (Biocytogen, Beijing, China) were used for orthotopic tumor xenograft assays. Approximately 1×10^6^ OSRC-2-lucifer cells stably expressing shCtrl, shFTO or SIK2 were resuspended in 20ul medium containing 2% fetal bovine serum and injected into the kidney capsule of B-NDG mice. When mice were 10 weeks old, they were treated with 3MA at 15 mg/kg/day (i.p.) for 7 consecutive days, and the control group was given vehicle PBS. For intravital imaging, the mice were anesthetized and injected with 150 mg/kg D-luciferin. Mice were placed into the IVIS-50 chamber (Caliper Life Sciences, USA) in the decubitus or supine position on the affected side. Emissions were collected using filters for green and background fluorescence, and Living Image software (Caliper Life Sciences, USA) was used to process the images. The ratio of fluorescent photon fluxes in the lesion area and the tumor-free background area was recorded, from which the size of the renal tumor was calculated.

### Patient-derived xenograft model (PDX model)

B-NDG (Biocytogen, Beijing, China) and BALB/c nude mice (Vital River Laboratory, USA) were used to construct the PDX ccRCC mouse model. Briefly, tumor tissues from two patients undergoing renal tumor resection at Peking University First Hospital were collected and stored at 4 °C in DMEM complete medium. Part of the tumor tissue was taken to make quick-frozen sections, which were identified as ccRCC by two senior urological pathologists. At the same time, tumor tissue was shredded to 1-3 mm^3^ and then implanted subcutaneously in the abdomen of B-NDG mice. When PDX tumors grew to 1-2 cm^3^, the tumor tissue was chopped and transplanted into the next generation of mice. The fourth generation of PDX-bearing mice started treatment with FE23-2 (MedChemExpress, USA) when tumors reached 50 mm^3^ as described previously 19. FB23-2 at 8 mg/kg/day (i.p.) was administered continuously for 14 days, and the control group was administered with vehicle DMSO. To ensure a consistent therapeutic dose of 6 mg/kg/day, mice were weighed daily during treatment and the dose received was recalculated. Mice were euthanized 6 weeks after the end of treatment, kidney tumors were dissected and collected for further analysis, and tumor volume and weight were recorded.

### Statistical analysis

Statistical analyses were performed using SPSS v23.0 (IBM Corp., Armonk, NY, USA) or GraphPad Prism v7.0 (GraphPad Software, La Jolla, CA, USA). Data are presented as the mean ± standard error of the mean (SEM). Univariate and multivariate Cox regression analyses were performed to examine independent factors, Pearson's test was used to assess correlation between different groups. A *p* value of < 0.05 was considered significant.

## Results

### m6A modification levels are decreased and closely correlated with autophagy in ccRCC

**Figure 1:**
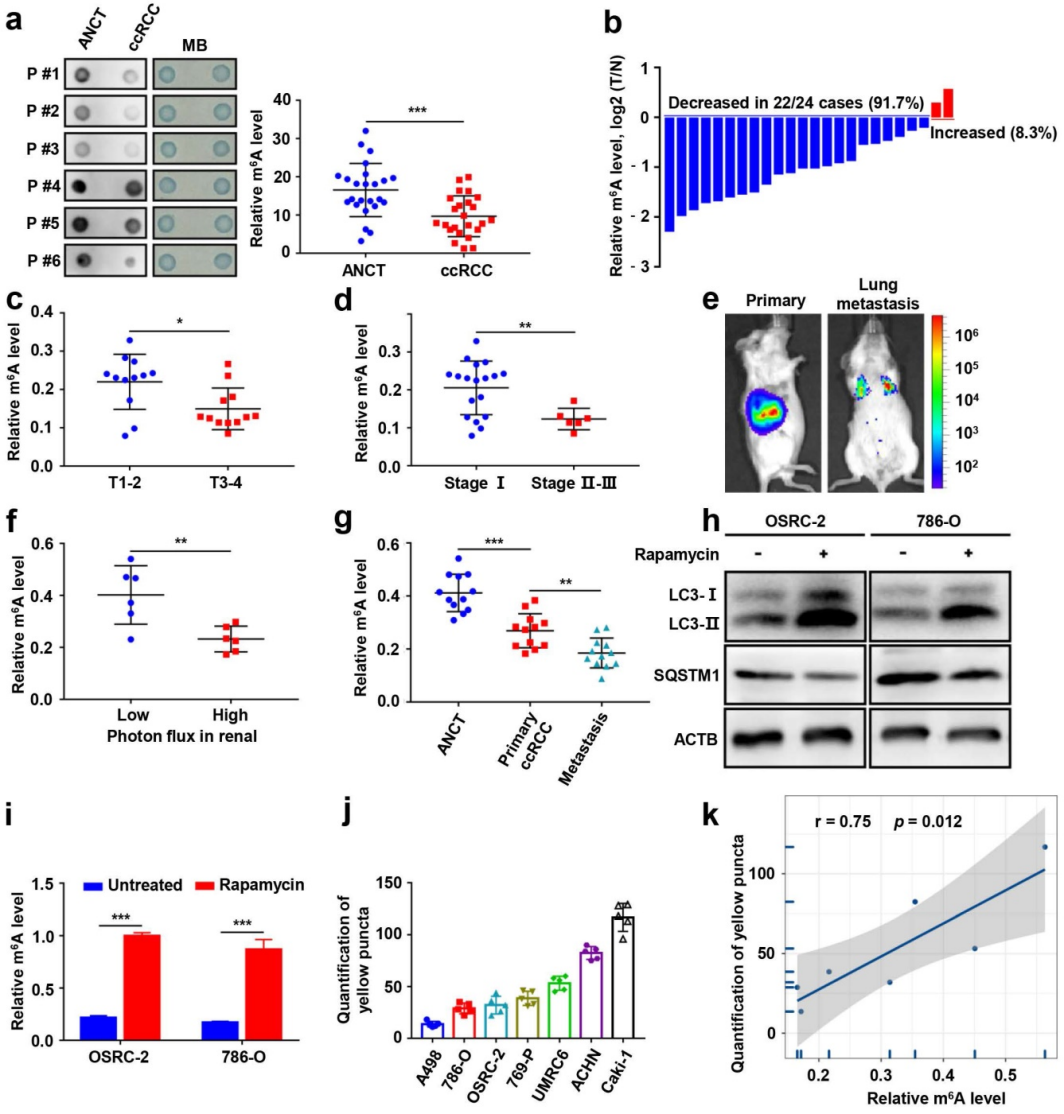
** m6A modification levels are decreased and closely correlated with autophagy in ccRCC. a)** The mRNAs isolated from ccRCC tissues and paired adjacent noncancerous tissues (ANCT) were used in dot blot analyses with an anti-m^6^A antibody, and MB (methylene blue) staining served as the loading control (representative images in the left panel), and the relative m^6^A RNA levels were calculated (right panel, n=24). **b)** The m^6^A RNA levels in the same 24 ccRCC tissues and paired ANCT were detected by the m^6^A RNA methylation quantification kit. **c)** The m^6^A RNA levels in T stage 1-2 versus T stage 3-4 of ccRCC tissues by the m^6^A RNA methylation quantification kit. **d)** The m^6^A RNA levels in stage I versus stage II-III of ccRCC tissues by the m^6^A RNA methylation quantification kit. **e)** Representative images of orthotopic transplantation and lung metastasis mouse model. **f)** Quantification of m^6^A levels in two groups with low and high photon flux of orthotopic tumor in mouse kidney by the m^6^A RNA methylation quantification kit. **g)** Quantification of m^6^A levels in ANCT, primary ccRCC, and lung metastases by the m^6^A RNA methylation quantification kit. **h-i)** LC3- I/II and SQSTM1 expression in OSRC-2 and 786-O cells treated with or without 100nM rapamycin for 72 hours using western blot analysis and the m^6^A levels in the total mRNA was determined using an m^6^A RNA methylation quantification kit. **j)** Quantification of LC3 puncta. **k)** Relationship between relative m^6^A RNA levels and autophagy in ccRCC cell lines. Error bars, SEM; *, *P* < 0.05; **, *P* < 0.01; ***, *P* < 0.001.

To determine the functional roles of m^6^A modification in ccRCC, we first measured the m^6^A RNA levels in 24 human ccRCC tissues and paired adjacent noncancerous tissues (ANCT). We found that the m^6^A levels were significantly lower in ccRCC than in ANCT via a dot blot assay (Fig. 1a). The results were also confirmed by the m^6^A RNA methylation quantification kit (Fig. 1b). Furthermore, we evaluated the relationship between m^6^A levels and clinicopathology and observed remarkably decreased m^6^A levels in T stage 3-4 than in T stage 1-2 (Fig. 1c), and the m^6^A levels were also decreased in stage II-III than in stage I (Fig. 1d). OSRC-2-luciferase cells were orthotopically injected into immunodeficient B-NDG mice (Fig. 1e), we found that m^6^A levels decreased in the higher photon flux group in the renal tumors (Fig. 1f), and they also decreased in lung metastases (Fig. 1g).

To analyze the correlation between autophagy and m^6^A RNA methylation in ccRCC, we established cellular models of rapamycin-induced autophagy. The m^6^A modification levels were significantly increased in the ccRCC cell lines OSRC-2 and 786-O after treatment with rapamycin (Fig. 1h-i). GFP signal is easily quenched in acidic autolysosomes, while RFP is more stable. Autophagosome and lysosome fusion occurs when GFP and mRFP fluorescence co-localize, resulting in the loss of yellow puncta and the appearance of only red puncta. Therefore, a green fluorescent protein (GFP)-monomeric red fluorescent protein (mRFP)-LC3B construct was used to determine the correlation between m^6^A RNA methylation and autophagic flux in ccRCC cell lines (Fig. S1, Fig. 1j). Correlation analysis revealed that autophagic flux and m^6^A levels were positively correlated in ccRCC cells (Fig. 1k). Together, these results suggest that m^6^A modification levels are significantly decreased and closely related to its autophagic flux in ccRCC.

### Elevated FTO expression correlates with poor prognosis of patients with ccRCC

**Figure 2:**
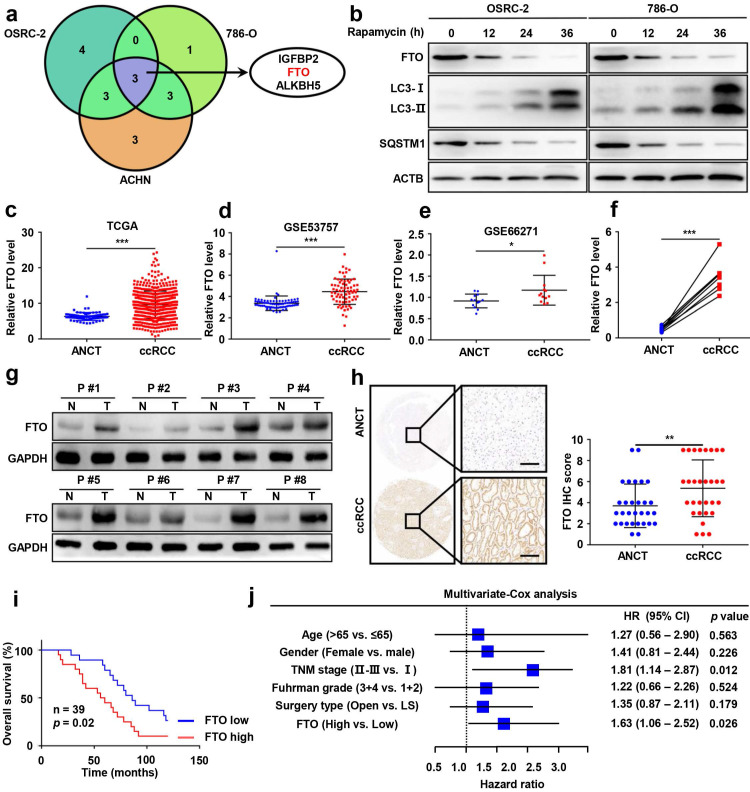
** Elevated FTO expression correlates with poor prognosis of patients with ccRCC. a)** A venn diagram of differentially expressed m^6^A-related genes in three ccRCC cell lines treated with 100nM rapamycin for 72 hours. **b)** FTO, LC3- I/II, and SQSTM1 expression in OSRC-2 and 786-O cells treated with rapamycin at 0, 12, 24, and 36 h, measured using western blot analysis.** c)** *FTO* mRNA expression in 539 ccRCC tumor tissues as compared to 72 adjacent noncancerous tissues (ANCT) from TCGA data. **d)** *FTO* mRNA expression in 72 paired ccRCC tumor tissues and ANCT from GSE53757 GEO datasets. **e)** *FTO* mRNA expression in 13 paired ccRCC tumor tissues and ANCT from GSE66271 GEO datasets. **f-g)** Real-time PCR and western blot analysis of *FTO* expression in 8 paired ccRCC tumor tissues and ANCT. **H)** Immunohistochemical (IHC) analysis of FTO in a ccRCC tissue microarray (TMA) 30 ccRCC tumor and ANCT pairs (left panel, scale bar, 100 µm) and quantification of FTO expression by IHC analysis in ccRCC TMA samples as compared to ANCT (right panel).** i)** Kaplan-Meier overall survival (OS) analysis of FTO expression in patients with ccRCC (log-rank test). **j)** Multivariable analyses were performed in the ccRCC cohort. All bars correspond to 95% CIs. Error bars, SEM; *, *P* < 0.05; **, *P* < 0.01; ***, *P* < 0.001.

To identify potential target genes of m^6^A RNA methylation in autophagy, we first employed qPCR analysis to compare the mRNA expression of m^6^A-related effectors (METTL3, METTL14, METTL16, WTAP, VIRMA, ZC3H13, RBM15, RBM15B, YTHDC1, YTHDC2, YTHDF1, YTHDF2, YTHDF3, HNRNPC, FMR1, LRPPRC, HNRNPA2B1, IGFBP1, IGFBP2, IGFBP3, RBMX, FTO, and ALKBH5) following rapamycin treatment. Our results showed that the mRNA levels of IGFBP2, FTO, and ALKBH5 were significantly attenuated after rapamycin treatment (Fig. 2a), while only FTO was also markedly downregulated at protein levels by rapamycin treatment (Fig. 2b and Fig. S2).

Having identified FTO as a potential target of m^6^A RNA methylation in autophagy, we sought to further assess FTO expression in ccRCC and ANCT. Bioinformatics analysis of 539 ccRCC samples and 72 adjacent noncancerous samples from The Cancer Genome Atlas (TCGA) database revealed significantly increased FTO expression in ccRCC tissues compared to ANCT (Fig. 2c). Subsequently, we analyzed two GEO datasets, GSE53757 and GSE66271, which also suggested that FTO is elevated in ccRCC compared to ANCT (Fig. 2d-e). Furthermore, we examined FTO expression in eight pairs of ccRCC tissues and ANCT, which further verified that FTO was significantly increased in ccRCC compared to ANCT at mRNA levels (Fig. 2f). In all eight pairs, the protein levels of FTO were found to be higher in human ccRCC tissues than in their ANCT by western blot (Fig. 2g). These results were confirmed in a ccRCC tissue microarray containing 30 pairs of ccRCC and adjacent tissue, which showed that FTO protein levels were increased in ccRCC tissue compared with that in ANCT by IHC staining (Fig. 2h). Additionally, the data showed that patients with high FTO expression had poorer overall 10-year survival than those with low FTO expression (n=39, p<0.05, log-rank test; Fig. 2i). Simultaneously, univariate and multivariate Cox regression revealed that high FTO expression was an independent prognostic factor for overall survival (OS) (HR=1.63, 95% CI: 1.06-2.52; p=0.026) in ccRCC (Fig. 2j). To further examine the predictive power of FTO expression levels in the OS of ccRCC patients, we performed a time-dependent receiver operating characteristic curve analysis and found that when FTO expression levels was combined with other clinical variables, its predictive efficiency was much better than either alone (Fig. S2). Taken together, these data suggest that FTO is elevated in ccRCC and may be related to autophagic flux in rapamycin-treated ccRCC cells, and its expression level might be an independent prognostic factor for patients with ccRCC.

### FTO downregulation enhances ccRCC autophagic flux by targeting ATG5 and ATG7

**Figure 3:**
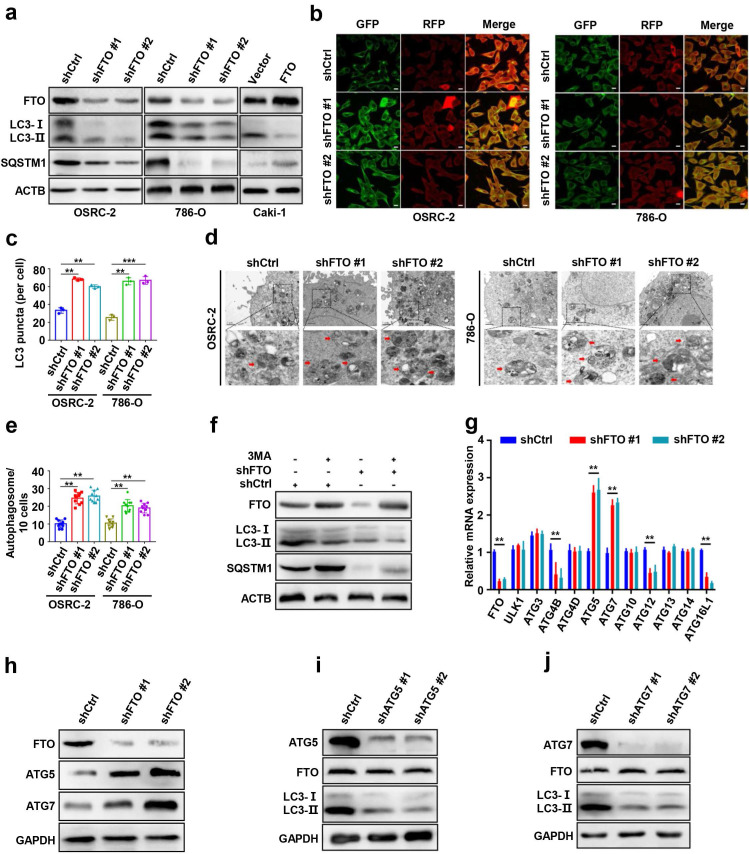
** FTO downregulation enhances ccRCC autophagic flux by targeting ATG5 and ATG7. a)** Plasmids containing FTO shRNA and FTO were transfected into OSRC-2, 786-O, or Caki-1 cells to establish stable cell lines. Western blot analysis was used to detect FTO, LC3- I/II, and SQSTM1 expression. **b-c)** IF staining with mRFP-GFP-LC3 in OSEC-2 and 786-O cells with FTO knockdown. Red puncta signify autolysosomes and yellow puncta signify autophagosomes and quantification of LC3 puncta. Scale bar, 10 µm. **d-e)** Transmission electron microscopy (TEM) demonstrating autophagosomes in OSRC-2 and 786-O cells with FTO knockdown, and quantification of autophagosomes in cells. Arrows indicate autophagosomes. Scale bar: 0.5 µm. **f)** Western blotting analysis of FTO, LC3- I/II, and SQSTM1 expression in control and FTO knockdown cells treated with or without 10 mM 3MA for 6h. **g)** qPCR analysis 0f expression levels of autophagy-related proteins in control and FTO knockdown cells. Actb was used as an internal control. **h)** Western blotting analysis of FTO, ATG5, and ATG7 expression in control and FTO knockdown cells. ACTB was used as a loading control. **i)** Western blotting analysis of ATG5, FTO, and LC3-I/II expression in control and ATG5 knockdown cells. **j)** Western blotting analysis of ATG7, FTO, and LC3-I/II expression in control and ATG7 knockdown cells. Error bars, SEM; *P* < 0.01; ***, *P* < 0.001.

To further explore the role of FTO in autophagy, we first silenced FTO in ccRCC cell lines using independent FTO short hairpin RNAs (shRNAs) in OSRC-2, and 786-O cells. The protein levels of microtubule-associated protein 1 light chain 3-beta (MAP1LC3B/LC3, an autophagy activation marker), and SQSTM1/p62 were measured by western blot to confirm that autophagy activation of cells had occurred (Fig. 3a). Silencing of FTO significantly increased the ratio of LC3-II: I and decreased SQSTM1 levels compered to control cells, suggesting a steady-state autophagosome formation (Fig. 3a). Immunofluorescence assays showed that more LC3 puncta were formed after FTO silencing in OSRC-2 and 786-O cells (Fig. 3b-c). Additionally, we also analyzed autophagosome formation by TEM and found that silencing of FTO increased the number of autophagosomes, indicating upregulated activation of autophagy (Fig. 3d-e). To confirm whether autophagy was affected by FTO deficiency, FTO-deficient and control cells were treated with or without 3-MA (an alternative autophagy inhibitor). We observed that 3-MA treatment of cells not only attenuated autophagy, but also increased FTO protein expression (Fig. 3f). These findings suggest that FTO negatively modulates autophagy activation in ccRCC cells.

To screen potential target genes of FTO in autophagy, we first compared the mRNA expression differences of autophagy-related genes following FTO deficiency by qPCR assays. The results showed that the mRNA levels of Atg5 and Atg7 were markedly increased following FTO deficiency (Fig. 3g). Consistently, FTO deficiency significantly upregulated ATG5 and ATG7 at protein levels (Fig. 3h). To further determine the upstream-downstream relationship between FTO and ATG5 and ATG7, we knocked down Atg5 and Atg7 in OSRC-2 cells, respectively. The knockdown efficiency was verified by western blot (Fig. 3i-j). The data showed that the LC3-II: I ratio markedly decreased following Atg5 and Atg7 depletion while FTO protein expression did not change, indicating that FTO was upstream of ATG5 and ATG7 (Fig. 3i-j). Collectively, these results confirmed that FTO downregulation may enhance autophagic flux by targeting ATG5 and ATG7.

### FTO downregulation impairs ccRCC growth *in vitro* and *in vivo*

**Figure 4:**
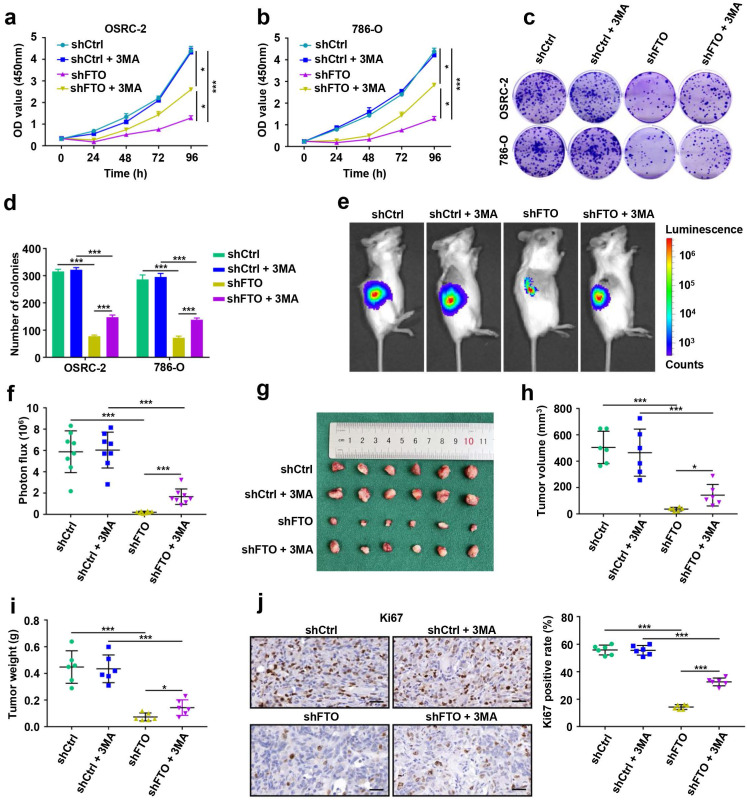
** FTO downregulation impairs ccRCC growth *in vitro* and *in vivo*. a-b)** Viability of OSRC-2 and 786-O cells (shCtrl and shFTO, treated with or without 10 mM 3MA) detected using the CCK8 assay. **c-d)** Representative images from the colony-forming assay and colony number analysis. All experiments were performed in triplicate and data were presented as the mean ± SD. **e)** Representative images of the orthotopic transplantation mouse model (shCtrl and shFTO, treated with or without 3MA). **f)** The analysis of the orthotopic tumor photon flux. **g)** Image of orthotopic transplanted tumors from the indicated groups. **h-i)** Tumors were removed 8 weeks after orthotopic transplantation, followed by volume calculation and weight measurement. **j)** Representative IHC staining images of Ki67 (scale bar, 100 µm, left panel) and quantification of Ki67 positive rate (right panel). Error bars, SEM; *, *P* < 0.05; ***, *P* < 0.001.

To examine the function of FTO in ccRCC, we conducted Cell Counting Kit-8 (CCK8) proliferation assays and 2D clonogenic assays in OSRC-2 and 786-O cells. FTO downregulation significantly attenuated the proliferation and 2D colony formation, whereas the inhibitory effect of silencing FTO on the malignant behavior of ccRCC cells was partially relieved by 3-MA treatment (Fig. 4a-d). Furthermore, we assessed the effect of FTO on ccRCC growth *in vivo*. OSRC-2-luciferase cells with knockdown of FTO and corresponding control cells were orthotopically injected into immunodeficient B-NDG mice. Tumor growth was impaired in FTO downregulation mice compared to WT mice, as illustrated by bioluminescence imaging (Fig. 4e). We observed that decreased FTO expression inhibited tumor growth (volume and weight, Fig. 4f-i) and Ki67 staining (Fig. 4j) in orthotopic transplantation models in B-NDG mice. Consistently, tumors showed decreased proliferation following FTO downregulation, but this inhibitory was partially relieved by 3-MA treatment (Fig. 4e-j). These data suggest that FTO deficiency impairs ccRCC growth *in vitro* and *in vivo*, and that this inhibitory function is partially alleviated by autophagy-inhibitory treatment.

### FTO downregulation impairs ccRCC metastasis *in vitro* and *in vivo*

**Figure 5:**
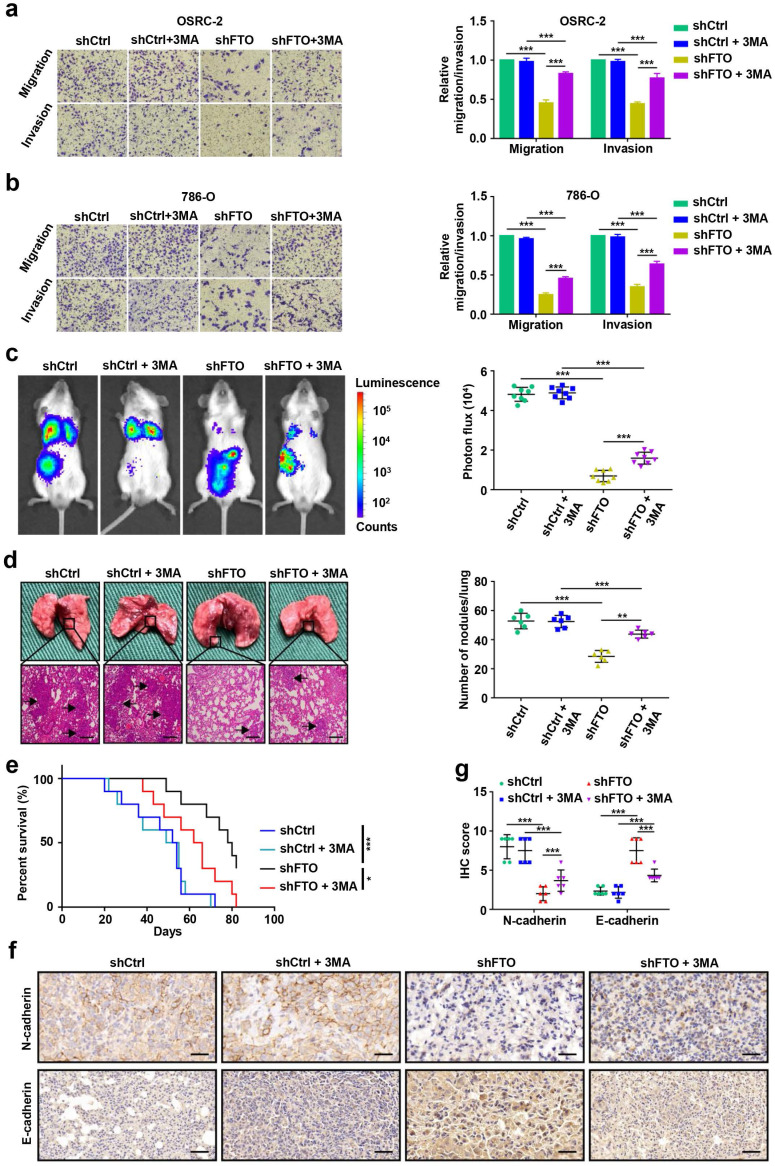
** FTO downregulation impairs ccRCC metastasis *in vitro* and *in vivo*. a-b)** Cell migration and invasion assays of OSRC-2 and 786-O cells after knockdown of FTO (treated with or without 10 mM 3MA). Representative images (left panel) and quantification (right panel) of the cell migration and invasion assay results were shown. **c)** Representative images of the orthotopic transplantation lung metastasis mouse model model (shCtrl and shFTO, treated with or without 3MA; left panel), and the analysis of the lung metastasis photon flux (right panel). **d)** Representative images of metastatic lung tumors and hematoxylin and eosin (H&E) staining (left panel), and quantification of lung tumors (right panel).** e)** The survival of mice orthotopically transplanted with control or knockdown FTO OSRC-2 cells was documented (n=10 mice per group). **f-g)** Representative IHC staining images of N-cadherin and E-cadherin (scale bar, 100 µm) and quantification of IHC score. Error bars, SEM; *, *P* < 0.05; **, *P* < 0.01; ***, *P* < 0.001.

To examine the roles of FTO in ccRCC metastasis, we first conducted migration and invasion assays *in vitro*. The results showed that downregulated expression of FTO impaired the migration and invasion of OSRC-2 and 786-O cells (Fig. 5a-b). Subsequently, we further evaluate the effect of FTO on ccRCC metastasis *in vivo*. After four weeks, downregulation of FTO significantly impairs ccRCC lung metastasis (Fig. 5c) and the number of lung metastatic lesions (Fig. 5d) compared with those in control groups, and this inhibitory was partially relieved by 3-MA treatment. Moreover, FTO knockdown in these ccRCC cells significantly prolonged the overall survival in mice with lung metastases (p=0.0004), and this effect was partially attenuated following 3-MA treatment (p= 0.0286, Fig. 5e). Compared with the control group, the N-cadherin IHC score decreased in the FTO downregulated group, whereas the E-cadherin IHC score increased, which were partially reversed by 3MA treatment (Fig. 5f-g). These data suggest that FTO deficiency impairs ccRCC metastasis *in vitro* and *in vivo*, and that this inhibitory function is partially alleviated by autophagy-inhibitory treatment.

### Identification SIK2 as the functional target of m^6^A-mediated autophagy in ccRCC cells

**Figure 6:**
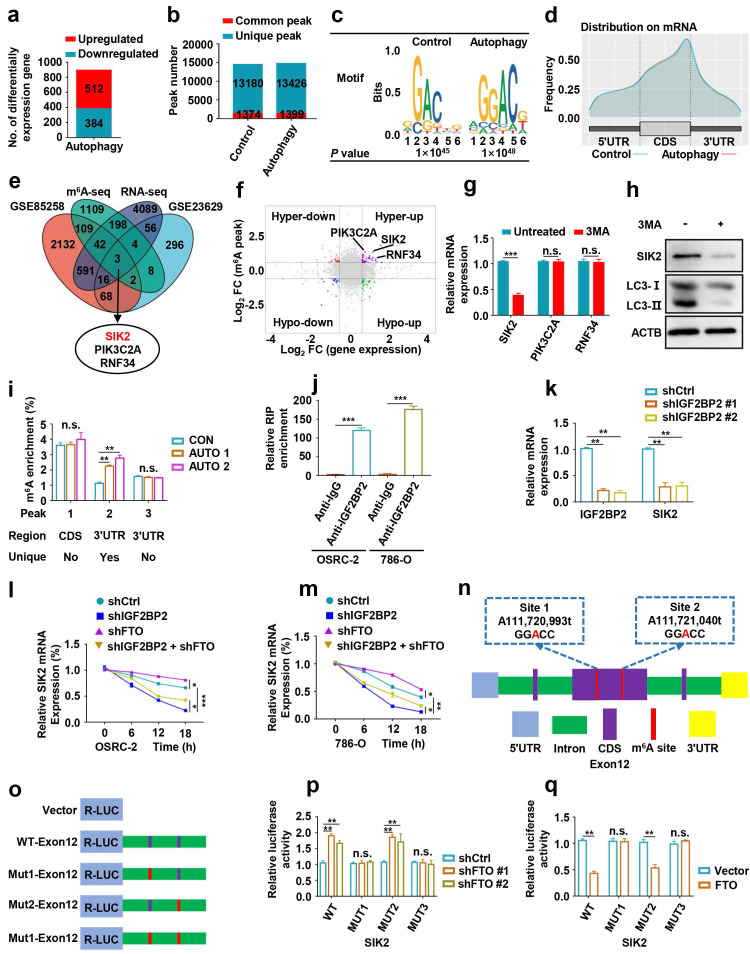
** Identification SIK2 as the functional target of m^6^A-mediated autophagy in ccRCC cells. a)** Differentially expressed genes with over 2-fold expression changes in OSRC-2 cells treated with or without 100nM rapamycin for 72 hours. **b)** Number of common and unique m^6^A peaks from MeRIP-seq. **c)** Top consensus motif identified from MeRIP-seq. **d)** Metagene profiles of m^6^A enrichment across mRNA transcriptome. **e)** A venn diagram of FTO-mediated autophagy downstream analysis.** f)** Localization of SIK2, PIK3C2A, and RNF34 in the quadrant chart of the distribution of peaks. **g)** SIK2, PIK3C2A, and RNF34 mRNA expression with or without 10 mM 3MA for 6h.** h)** Western blotting analysis of SIK2 and LC3- I/II expression in control and FTO knockdown cells treated with or without 10 mM 3MA for 6h.** i)** MeRIP-qPCR analysis of SIK2 RNA from OSRC-2 cells (treated with or with without rapamycin). **j)** RNA immunoprecipitation (RIP)-qPCR assay using IGF2BP2-specific antibody and IgG control antibody to detect the enrichment of IGF2BP2 binding to SIK2 m^6^A modification sites. **k)** qPCR analysis of SIK2 and IGF2BP2 in OSRC-2 cells (control and IGF2BP2 depleted). **l-m)** RNA stability analysis of SIK2 mRNA levels in OSRC-2 and 786-O cells (control or IGF2BP2 depleted) in the absence or presence of FTO silencing, and after actinomycin D treatment. **n)** Location of the m^6^A site in the *SIK2* mRNA sequence.** o)** Schematic diagram of dual-luciferase reporter constructs. **p-q)** Relative luciferase activity of WT or MUT (A-to-T mutation) *SIK2* luciferase reporter in control, FTO knockdown and FTO overexpressing cells. Error bars, SEM; *, *P* < 0.05; **, *P* < 0.01; ***, *P* < 0.001.

To identify potential mRNA targets of m^6^A modification, OSRC-2 cells undergoing rapamycin-induced autophagy were selected for methylated RNA immunoprecipitation sequencing (MeRIP-seq) and RNA sequencing (RNA-seq). The data showed that 512 genes were upregulated and 384 genes were downregulated after rapamycin-induced autophagy (Fig. 6a). MeRIP-seq identified 14554 and 14825 m^6^A peaks, of which 1374 and 1399 were unique in the control cells and cells with autophagy induction, respectively (Fig. 6b). Consistent with previous studies 20, the most common m^6^A motif, GGAC was highly enriched in the m^6^A peaks (Fig. 6c). Notably, these m^6^A modification were mainly enriched in the coding region (CDS) and 3′-untranslated region (3′-UTR) (Fig. 6d, Fig. S3). Gene Ontology and Kyoto Encyclopedia of Genes and Genomes pathway analyses demonstrated that the differentially expressed genes were associated with biological adhesion, biological regulation, growth, environment adaptation, and nucleotide metabolism (Fig. S4). To identify potential targets of m^6^A in the regulation of autophagy, we comprehensively analyzed RNA-seq and MeRIP-seq data. We compared genes with differential expression and m^6^A enrichment in our MeRIP-seq and RNA-seq datasets from the control and cells undergoing rapamycin-induced autophagy, and with differential expression of human matched primary ccRCC and metastasis (GSE85258 21 and GSE23629 22), generated from previous studies. We observed that three genes SIK2, PIK3C2A, and RNF34, appeared in the overlapping region of the above four datasets (Fig. 6e). In addition, SIK2, PIK3C2A and RNF34 were hypermethylated-upregulated (Fig. 6f). Furthermore, we examined the mRNA levels of SIK2, PIK3C2A, and RNF34 in 3-MA treated OSRC-2 cells and found that SIK2 was markedly upregulated, while PIK3C2A and RNF34 were not changed (Fig. 6g). Consistently, SIK2 protein levels decreased following 3-MA treatment (Fig. 6h). Our MeRIP-seq data revealed that the m^6^A abundance in the SIK2 mRNA was markedly increased during autophagy flux (Fig. S5). To further validate m^6^A methylation in SIK2 mRNA, we conducted methylated RNA immunoprecipitation quantitative PCR (MeRIP-qPCR). As expected, MeRIP-qPCR assay analysis showed that the m^6^A modification level of SIK2 mRNA was significantly increased after autophagy activation (Fig. 6i).

Studies have shown that m^6^A methylation modification on mRNA affect mRNA stability, which is mediated by specific m^6^A-binding proteins 8, 23, 24. As a main m^6^A “reader”, IGF2BP2 (Insulin-like growth factor 2 mRNA-binding protein 2) has been reported to promote the tumor progression by selectively recognizing and stabilizing m^6^A-modified mRNA 25. The IGF2BP2-specific antibody markedly enriched SIK2 mRNA in the RNA immunoprecipitation-qPCR assay compared to the IgG control antibody, indicating that IGF2BP2 binds SIK2 mRNA (Fig. 6j). To determine whether IGF2BP2 is a potential reader for SIK2 m^6^A methylation, we knocked down IGF2BP2 and observed strongly decreased expression of SIK2 in ccRCC cells (Fig. 6k). IGF2BP2 knockdown not only increased SIK2 mRNA stability and expression levels, but also abolished the attenuation caused by FTO silencing when the transcription was blocked with actinomycin D (Fig. 6l-m).

Next, we assessed whether FTO and IGF2BP2-mediated regulation is necessary for m^6^A modification on target mRNAs. Among the m^6^A peaks in our MeRIP-seq dataset, the multiple m^6^A peaks in exon 12 are more consistent and contain two GGAC motifs (Fig. 6n). We performed dual luciferase reporter and mutagenesis assays and generated five luciferase reporters including Renilla luciferase, wild-type, mutant 1, mutant 2, and mutant 3 of SIK2 exon2 (A was replaced by T in GGAC) (Fig. 6o). The luciferase assays revealed that FTO downregulation could increase the luciferase activity of the reporter construct bearing wild-type and mutant 2, but not mutant 1 and mutant 3 (Fig. 6p). Consistent results were observed when cells were co-transfected with plasmids overexpressing FTO and luciferase (Fig. 6q). Together, our findings indicate that FTO-mediated m^6^A modification regulates SIK2 expression via IGF2BP2-dependent SIK2 mRNA stability.

### Effects of FTO-mediated SIK2 on autophagic flux, progression and survival in ccRCC

**Figure 7:**
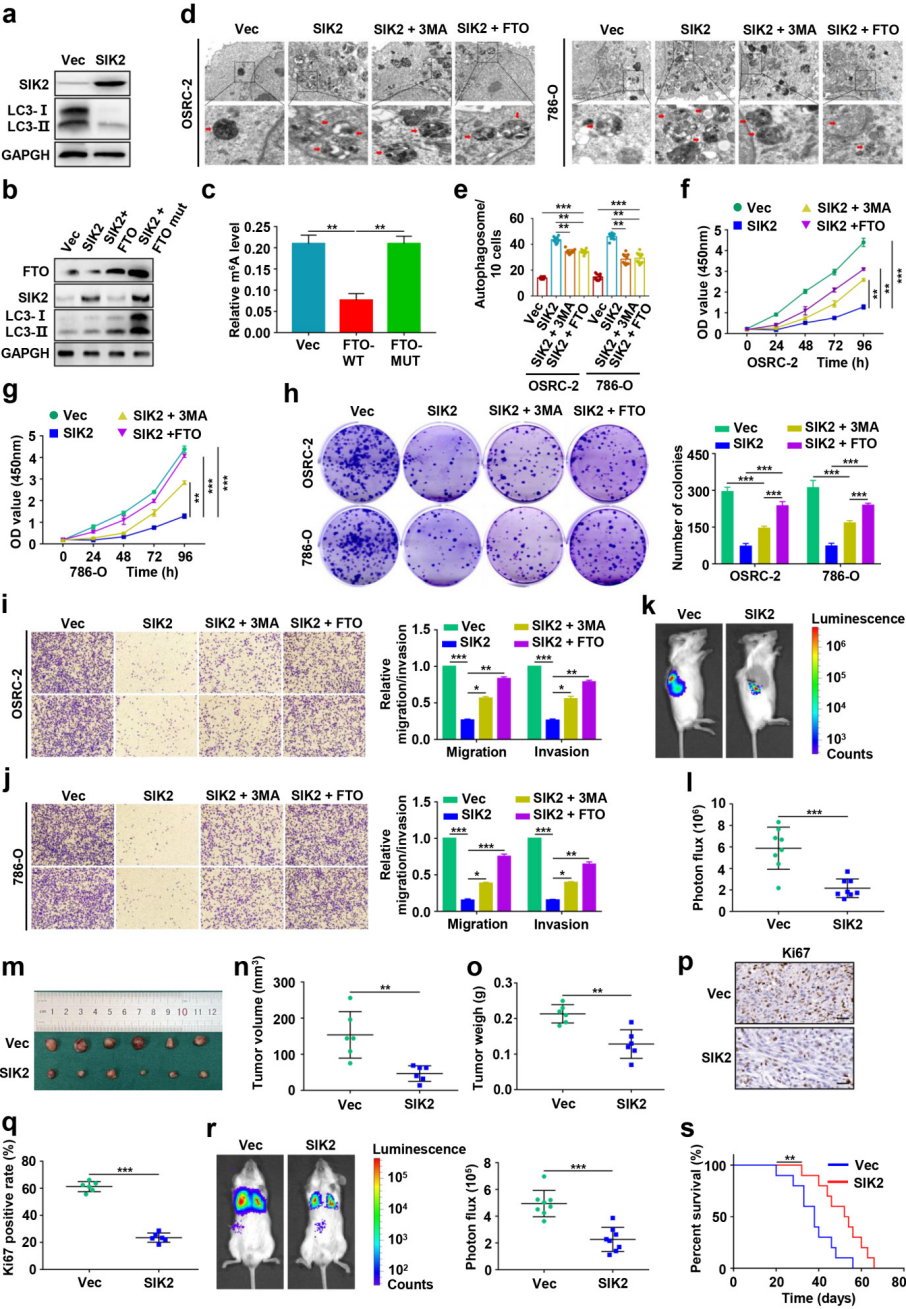
** Effects of FTO-mediated SIK2 on autophagic flux, progression and survival in ccRCC. a)** Western blot analysis was used to detect SIK2 and LC3- I/II expression in control and SIK2 overexpressing cells. **b)** Western blot analysis of FTO, SIK2, and LC3- I/II expression in control and SIK2 overexpressed OSRC-2 cells with FTO-WT or FTO-MUT overexpression. **c)** Quantification of m^6^A levels in vector, FTO-WT, and FTO-MUT by the m^6^A RNA methylation quantification kit. **d)** TEM demonstrating autophagosomes in control and SIK2 overexpressing OSRC-2 and 786-O cells with 3MA treatment or FTO overexpression. Arrows indicate autophagosomes. Scale bar: 0.5 µm. **e)** Quantification of autophagosomes in cells. **f-g)** Viability of OSRC-2 and 786-O cells (vector and SIK2, treated with 10 mM 3MA or FTO overexpression) detected using the CCK8 assay.** h)** Representative images from the colony-forming assay (left panel) and colony number analysis (right panel). All experiments were performed in triplicate and data were presented as the mean ± SD.** i-j)** Cell migration and invasion assays of OSRC-2 and 786-O cells after overexpression of FTO (treated with 10 mM 3MA or FTO overexpression). Representative images (left panel) and quantification of the cell migration and invasion assay results (right panel) were shown. **k)** Representative images of the orthotopic transplantation mouse model (vector and SIK2). **l)** The analysis of the orthotopic tumor photon flux. **m)** Image of orthotopic transplanted tumors from the indicated groups. **n-o)** Tumors were removed 8 weeks after orthotopic transplantation, followed by volume calculation and weight measurement.** p-q)** Representative IHC staining images of Ki67 (scale bar, 100 µm) and quantification of Ki67 positive rate. **r)** Representative images of the orthotopic transplantation lung metastasis mouse model model (vector and SIK2; left panel), and the analysis of the lung metastasis photon flux (right panel). **s)** The survival of mice orthotopically transplanted with control or SIK2 overexpressed OSRC-2 cells was documented (n=10 mice per group). Error bars, SEM; *, *P* < 0.05; **, *P* < 0.01; ***, *P* < 0.001.

To explore the role of SIK2 in ccRCC, we overexpressed SIK2 and found that autophagic flux was enhanced, suggesting that autophagy was activated (Fig. 7a-b). To further clarify the underlying molecular mechanism by which FTO regulates autophagy, we constructed the wild-type (FTO-WT) and catalytic mutant FTO^R96Q^ (FTO-MUT) plasmids to determine whether the demethylase activity of FTO was required 8, 26. The effect of ectopic expression of FTO-WT or FTO-MUT on the total cellular m^6^A level was confirmed by the m^6^A RNA methylation quantification kit (Fig. 7b-c). Upregulation of FTO-WT reversed SIK2 expression and increased LC3-II: I ratio, but FTO-MUT failed for both, indicating that the demethylase activity of FTO is involved in this mechanism (Fig. 7b). Furthermore, we analyzed autophagosome formation by TEM and found that SIK2 upregulation increased the number of autophagosomes, but the ectopic expression FTO or 3-MA treatment reversed the enhanced autophagy (Fig. 7d-e).

Moreover, overexpression of SIK2 in OSRC-2 and 786-O cells resulted in decreased in both proliferation (Fig. 7f-h), migration, and invasion (Fig. 7i-j). However, 3-MA treatment or FTO upregulation partially reversed these effects. Results of orthotopic injected ccRCC models showed significantly lower photon flux of tumors in the SIK2 overexpression group at the third week compared to the control group (Fig. 7k-l). Noticeably, SIK2 overexpression group mice had smaller tumor volume (Fig. 7m-n) and tumor weight (Fig. 7o) with decreased Ki67 staining compared with control mice (Fig. 7p-q). Consistently, SIk2 overexpression group mice had fewer lung metastasis photon flux (Fig. 7r) and the number of lung metastatic nodules (Fig. S6) compared with those in control groups. As expected, overexpression of SIK2 markedly prolonged overall survival in ccRCC mice (Fig. S7). Compared with the control group, the N-cadherin IHC score decreased in the SIK2 ectopic expression group, whereas the E-cadherin IHC score increased (Fig. S7). Together, these results demonstrate that FTO-mediated SIK2 can promote autophagic flux and the enforced expression of SIK2 efficiently inhibits the progression and survival of ccRCC *in vitro* and *in vivo*.

### Small-molecule inhibitor targeting FTO exhibits therapeutic efficacy in a PDX ccRCC mouse model

**Figure 8:**
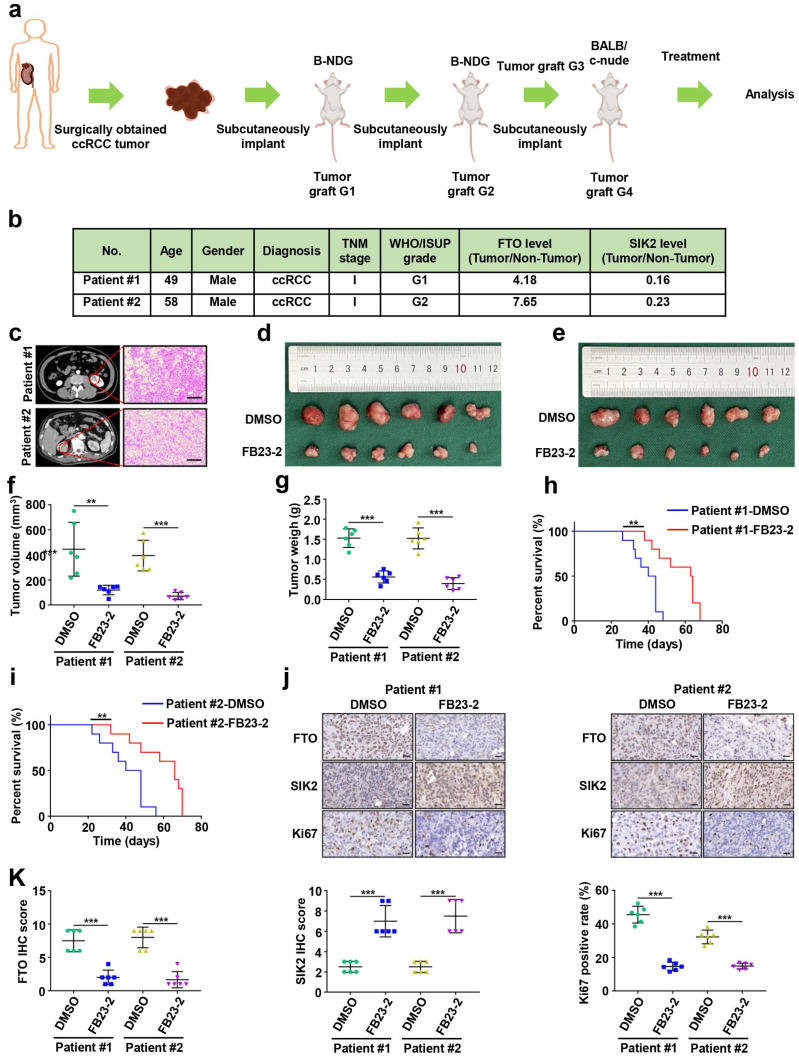
** Small-molecule inhibitor targeting FTO exhibits therapeutic efficacy in a patient-derived xenograft (PDX) ccRCC mouse model. a)** Graphic illustration of ccRCC PDX mouse models. **b)** Clinical characteristics of the donor patients.** c)** Computer tomography (CT) scan and H&E staining of donor patients. Representative images of CT scan (left panel) and H&E staining (right panel). **d-e)** Harvested PDX tumors in the control and FB23-2 treatment groups. **f**) Tumor volume of the PDX tumors. **g)** Tumor weight of the PDX tumors. **h-i)** The survival of PDX mice treated with DMSO or FB23-2 was documented (n=10 mice per group). **j-k)** Representative IHC staining images of FTO, SIK2, and Ki67 (scale bar, 100 µm) and quantification of IHC score. Error bars, SEM; **, *P* < 0.01; ***, *P* < 0.001.

Several FTO small-molecule inhibitors such as FB23-2 6, Dac51 27, R-2HG 28, and CS1/CS2 29 have been reported to inhibit the demethylation activities of FTO. Recently, a study showed that the FTO small-molecule inhibitor FB23-2 directly binds FTO and selectively inhibits m6A demethylase activity 6. More importantly, FB23-2 dramatically inhibited the progression of human acute myeloid leukemia cell lines and primary cells in xenografted mice 6, highlighting the broad potential of targeting FTO for cancer therapy. However, the anticancer effect of FB23-2 on solid tumors including ccRCC is unclear. Therefore, we applied FB23-2 in the PDX model to validate whether FTO is a druggable target of ccRCC. We generated ccRCC PDX murine models (Fig. 8a), and the clinicopathological characteristics of the donor patients are shown in Fig. 8b-c. Results revealed that mice in the FB23-2-treated group had smaller tumor volume and tumor weight (Fig. 8d-g), as well as a longer survival benefit (Fig. 8h-i), compared to control mice. IHC staining confirmed decreased FTO expression in the FB23-2 treatment group and increased SIK2 expression in the control group (Fig. 8j-k). The Ki67 expression level was dramatically decreased in the FB23-2 treated group compared with the control group (Fig. 8j-k). Collectively, our data suggest that FTO is a druggable target and that FB23-2, a small molecule inhibitor targeting FTO, have the potential to treat ccRCC.

**Figure 9:**
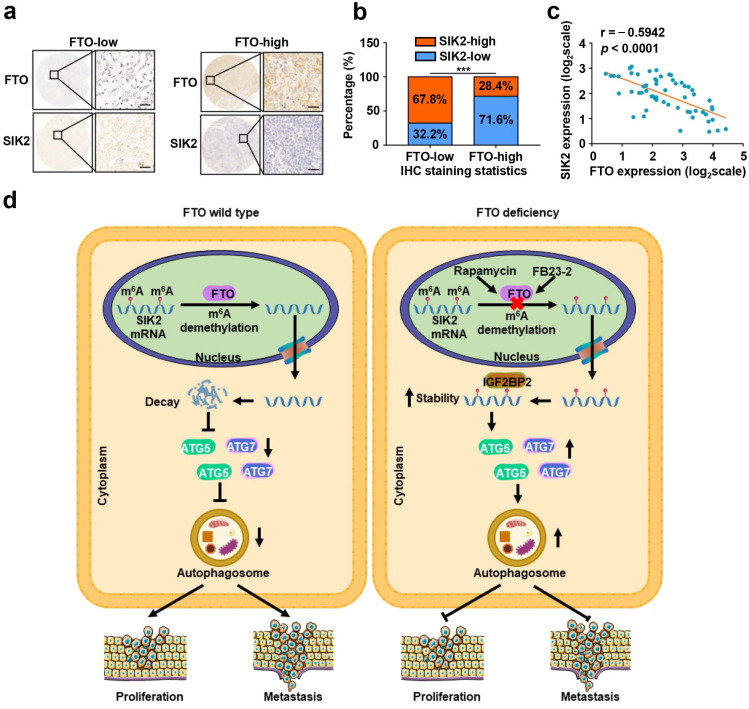
** FTO and SIK2 expression is negatively correlated. a)** Representative IHC staining images of FTO with different dyeing intensity and corresponding SIK2 staining were shown, respectively (scale bar, 100 µm). **b)** Statistics of IHC staining displayed the percentages of ccRCC specimens with lower or higher FTO expression and corresponding SIK2 levels. **c)** qPCR analysis revealed a negative correlation between FTO and SIK2 expression. **d)** The graphic illustration of FTO-mediated autophagy promoting the progression of ccRCC. Error bars, SEM; *P* < 0.001.

To further assess the clinical significance of FTO-SIK2-autophagy axis promoting ccRCC progression, we examined the expression of FTO and SIK2 in tumor tissues of patients with ccRCC and subsequently categorized the samples into FTO-low and FTO-high groups and determined their relevance in ccRCC. The expression of FTO was negatively correlated with the expression of SIK2 in 60 ccRCC patients (Fig. 9a-b), and the validation of mRNA levels also supported this correlation (Fig. 9c).

## Discussion

RNA m^6^A modification has been implicated in the tumorigenesis, progression, and therapeutic response of various tumors, but potent inhibitors targeting m^6^A regulators are still under development. FTO, one of the key demethylases, has been found to play an oncogenic role in leukemia 29 and oral squamous cell carcinoma 15, and may be a potential therapeutic target. However, the possible role of FTO in ccRCC tumor progression remains unknown. In the present study, we revealed that FTO is an oncogene of ccRCC that mediates autophagy to promote ccRCC progression through the IGF2BP2-SIK2 pathway. More importantly, we validated that the small molecule inhibitor FB23-2 targeting FTO could significantly inhibit tumor growth and prolong the survival time of mice in the PDX model analysis. At the molecular level, rapamycin-induced autophagy attenuates FTO expression at mRNA and protein levels, while silencing FTO suppresses autophagic flux by targeting ATG5 and ATG7. Furthermore, we identified SIK2 as a critical downstream target of FTO-mediated autophagy, whose mRNA stability is regulated by m^6^A modification through m^6^A reader IGF2BP2 (Fig. 9d). Collectively, these results establish that FTO-autophagy interaction is an epitranscriptional mechanism of ccRCC tumor progression. Our findings are highly significant as they identify for the first time the tumor suppressive role of a small molecule inhibitor targeting FTO in solid tumors and establish a key pathway by which m^6^A modification mediates autophagic flux to modulate tumor progression.

Since the concept of autophagy was introduced by Christian de Duve in 1963 30, great progress has been made in understanding how autophagy plays a role in physiological and pathological metabolic functions and contributes to improved clinical outcomes. Autophagy occurs at the basal level in all cells and is induced by multiple signals and cellular stresses. Autophagy plays a dual role in the regulation of cancer. On the one hand, it prevents the occurrence of cancer cells, but on the other hand, it is a key mechanism for the survival of cancer cells. Growing evidence suggests that dysregulation of autophagy triggers oncogenic mutations, organelle damage, and accumulation of harmful metabolic molecules that induce oxidative stress, genomic instability, DNA damage, inflammation, and tissue damage, all of which are tumorigenesis inducers 14. Consistent with the finding of the present study, autophagy was downregulated in multiple types of cancers, suggesting that it has a tumor suppressor role. There are various proteins involved in autophagy regulation, such as Atg4c 31, DAP kinase 32, and PTEN 33, have shown their role in tumor suppression. As a canonical survival pathway, the PI3K-Akt-mTOR signaling axis is activated in various cancers to promote tumor cell proliferation and survival, and the constitutive activation of this axis inhibits autophagy 34. Thus, identifying therapeutic targets for autophagy in specific tumors is critical for improving patient outcomes.

The regulation of autophagy by m^6^A is dual, m^6^A modification promotes autophagy under certain conditions, but inhibits autophagy activity under other conditions. Recently, studies have demonstrated a close mechanistic correlation between m^6^A RNA methylation and autophagy in tumorigenesis, progression, and response to therapy. The m^6^A methyltransferase METTL13 positively regulates autophagic flux by upregulating the expression of LC3B, ATG5, and ATG7 and plays a key role in β-elemene reversal of gefitinib resistance in non-small cell lung cancer 35. Li et al. revealed that HIF-1α-induced YTHDF1 expression promotes the translation of autophagy-related genes ATG2 and ATG14 in an m^6^A-dependent manner, thereby promoting the progression of hepatocellular carcinoma 19. These findings undoubtedly greatly increase the understanding of the regulation of tumor cell mechanisms and provide novel ideas for the development of potential therapeutic targets.

Importantly, the m^6^A demethylase FTO has been identified as an essential regulator of m^6^A-autophagy interactions in tumorigenesis. In arsenic-related human skin injury, arsenic stabilizes FTO expression by attenuating p62-mediated selective autophagy, and the upregulation of FTO in turn impairs autophagy, leading to a positive feedback loop that maintains FTO accumulation and induce malignant transformation and tumorigenesis 36. In human melanoma, metabolic starvation stress regulated by autophagy induces the upregulation of FTO, which in turn promotes tumorigenesis 37. FTO upregulates ULK1 protein abundance and activates autophagy by reversing m6A modification in the 3′-UTR region of ULK1 mRNA, leading to autophagy-mediated tumorigenesis 38. In epithelial ovarian cancer, FTO binding to circRAB11FIP1 leads to its upregulation, and the induced autophagy accelerates tumorigenesis and progression 39. Furthermore, our results revealed that rapamycin-induced autophagy activation reduced FTO protein abundance, and FTO-mediated autophagy greatly promoted tumor progression including lung metastasis. These findings highlight the interplay of FTO-mediated mRNA m^6^A modification and autophagy, broadening the multilevel regulation of m^6^A modification in tumor evolution, which is critical for effective therapeutic applications. Our findings are consistent with previous studies confirming that FTO is an oncogene in ccRCC 40. Overexpressed FTO in ccRCC was identified as a synthetic lethal partner of tumor suppressor gene von Hippel-Lindau (*VHL*) 40, which was also supported by a recent study demonstrating that FTO depletion inhibited ccRCC cells proliferation 41. However, there was also a report suggesting that FTO made it a tumor suppressor in ccRCC by blocking the FTO-PGC-1α signaling axis 42. Elimination of this controversy may need to be clarified in the homogenization model.

To explore the underlying mechanism of m^6^A demethylase FTO-mediated autophagy in ccRCC, we identified SIK2 as a key regulator that plays a decisive role in malignant tumor proliferation and distant metastasis including lung metastasis. Salt-inducible kinase (SIK) was first identified in the adrenal cortex of rats with a high-salt diet, and its three isoforms (SIK1, SIK2, and SIK3) belong to the AMP-activated protein kinases (AMPKs) family. Among them, SIK2 encodes a protein containing 931 amino acids, and its kinase domain is located in the 20-271 residue region 43. SIK2 has been reported to be involved in multiple biological roles including malignant progression of ovarian cancer 44, neuronal survival after ischemia 45, and the restriction of intracellular *Salmonella* proliferation 46. In many human malignancies, SIK2 exhibits significant differential expression, and it plays a key regulatory role involving the PI3K/AKT, AKT/GSK3β/β-catenin, Hippo-YAP, LKB1-HDAC, and cAMP-PKA pathway. Gao et al. revealed that SIK2 upregulates HIF-1α and promotes glycolysis by activating the PI3K/AKT signaling pathway in ovarian cancer 47, and SIK2-mediated reprogramming of glucose metabolism enhances ovarian cancer cell proliferation and attenuates apoptosis. Dai et al. found that SIK2 was significantly down-regulated in gastric cancer and inhibited epithelial-mesenchymal transition by inhibiting the AKT/GSK3β/β-catenin signaling pathway 48. In addition, the results from Rong et al. suggested that SIK2 expression was positively correlated with breast cancer recurrence, and SIK2 directly phosphorylated LRP6 resulting in activation of Wnt/β-catenin signaling pathway 49. However, it is unclear whether SIK2 plays a role in the biological function of ccRCC. Thus, our findings have revealed that SIK2 mRNA stability is regulated by IGF2BP2-dependent pathway, resulting in a regulatory role in FTO-mediated autophagy-related ccRCC malignant progression.

In summary, our results reveal m^6^A modification levels are decreased and closely related to autophagic flux in ccRCC, and we also found that elevated expression of FTO is inhibited by rapamycin treatment, and silencing FTO impairs ccRCC growth and metastasis. Mechanistically, we first identified SIK2 as a functional target of m^6^A-mediated autophagy, thereby prompting FTO to play a conserved and important role in inhibiting autophagy and promoting tumorigenesis through an m^6^A-IGF2BP2 dependent mechanism. Moreover, the small molecule inhibitor FB23-2 targeting FTO inhibited tumor growth in the PDX, suggesting that FTO is a potential therapeutic target for ccRCC. Our findings demonstrate that FTO could be a critical prognostic biomarker and promising therapeutic target in ccRCC.

## Supplementary Material

Supplementary figures and tables.

## Abbreviations

ccRCC: clear cell renal cell carcinoma
m^6^A: N^6^-methyladenosine
FTO: fat mass and obesity-associated protein
PDX: patient-derived xenograft
TMA: tissue microarray
qRT-PCR: quantitative real-time PCR
ATCC: American Type Culture Collection
PBS: phosphate buffered saline
H&E: hematoxylin and eosin
IHC: immunohistochemistry
TEM: transmission electron microscopy
RIP: RNA immunoprecipitation
ANCT: adjacent noncancerous tissue
GFP: green fluorescent protein
mRFP: monomeric red fluorescent protein
TCGA: The Cancer Genome Atlas
OS: overall survival
shRNAs: short hairpin RNAs
MeRIP-seq: methylated RNA immunoprecipitation sequencing
RNA-seq: RNA sequencing
IGF2BP2: insulin-like growth factor 2 mRNA-binding protein 2
