# Supplementary material for: FTO-mediated autophagy promotes progression of clear cell renal cell carcinoma via regulating SIK2 mRNA stability
Source: Int J Biol Sci. 2022 Oct 3;18(15):5943–62. doi: 10.7150/ijbs.77774 (PMC9576516; doi:10.7150/ijbs.77774)
Supplement: Supplementary file 1 — Supplementary figures and tables. [file ijbsv18p5943s1.pdf]

Supplementary Material

FTO-mediated autophagy promotes progression of clear cell renal cell carcinoma via regulating SIK2 mRNA stability

Table S1. Sequences of primers.

Table S2. Primary antibodies.

Figure S1. Immunofluorescence staining with mRFP-GFP-LC3 in ccRCC cell lines.

Figure S2. IGFBP2 and ALKBH5 expression in OSRC-2 and 786-O cells treated with rapamycin.

Figure S3. The time-dependent receiver operating characteristic (ROC) analysis for the FTO risk score, the clinical variables risk score, and the combined FTO and clinical variables risk scores in the ccRCC cohort.

Figure S4. m^6A peak distribution in the 5′UTR, 3′UTR, and CDS across the entire set of mRNA transcripts.

Figure S5. MeRIP-seq data revealed that m^6A abundance in the SIK2 mRNA was markedly increased during autophagy flux.

Figure S6. Gene Ontology pathway and Kyoto Encyclopedia of Genes and Genomes pathway analyses of differentially expressed genes in autophagy-induced OSRC-2 cells compared with normal OSRC-2 cells.

Figure S7. Representative images of metastatic lung tumors and hematoxylin and eosin staining, and quantification of lung tumors.

Figure S8. Representative IHC staining images of N-cadherin and E-cadherin and quantification of IHC score.

Table S1. Sequences of primers

Gene Symbol	Sequences
FTO	Forward 5′- ATTCTATCAGCAGTGGCAGC -3′<br>Reverse 5′- GGATGCGAGATACCGGAGTG -3′
ULK1	Forward 5′- CCTCGGTCAGGAAATCCCCA -3′<br>Reverse 5′- GAGCTGTGCAGGAAGCCA -3′
ATG3	Forward 5′- CCCCAGGATGCAGAATGTGA -3′<br>Reverse 5′- TCCAGCTGCCACAAACTCTT -3′
ATG4B	Forward 5′- TCCATCGCTGTGGGGTTTTT -3′<br>Reverse 5′- TCCAGTCGCTCTACATCAGA -3′
ATG4D	Forward 5′- ACACATCCTCAGGAAAGCCG -3′<br>Reverse 5′- TGACCACAGACTTCCACTCG-3′
ATG5	Forward 5′- GCAACTCTGGATGGGATTGC -3′<br>Reverse 5′- AGGTCTTTCAGTCGTTGTCTGAT -3′
ATG7	Forward 5′- AGCATCCAGAAGGGGGCTA -3′<br>Reverse 5′- TGCAGCAATGTAAGACCAGTCA -3′
ATG10	Forward 5′- ACATTCCAACGTTATTGTGCAG -3′<br>Reverse 5′- GCTCGAAAGCCTCCTCCAT -3′
ATG12	Forward 5′- TGCTGGAGGGGAAGGACTTA -3′<br>Reverse 5′- CCATCACTGCCAAAACACTCA -3′
ATG13	Forward 5′- AGCCCGGAACCACTCTT -3′<br>Reverse 5′- TCACTTGGACAGTCTTGAGGG -3′
ATG14	Forward 5′- TACGTGGCTGTGGAGCG -3′<br>Reverse 5′- CCTTCCATAGCTTTTAACACTTCTT -3′
ATG16L1	Forward 5′- TTGGCCCAGAAACTACAGGC -3′<br>Reverse 5′- CCAGTTGAGCTAACTCCCCA -3′
SIK2	Forward 5′- CTGAAGCCAGGCGAAAATTCT -3′<br>Reverse 5′- TTGCCAGCAGTTCACCACTTT -3′
PIK3C2A	Forward 5′- GGTTCCGATCCTCTGCGTT -3′<br>Reverse 5′- GGTTCCGATCCTCTGCGTT -3′
RNF34	Forward 5′- GGAGAGCTTATGGATGGAGACC -3′<br>Reverse 5′- GGTTCCGATCCTCTGCGTT -3′
IGF2BP1	Forward 5′- TTGGGACGCCATCAGTACCTA -3′<br>Reverse 5′- TTGGCTAAACTCTCTACGACTCT -3′
IGF2BP2	Forward 5′- TGCACATCCCCAACTGTGAC -3′<br>Reverse 5′- TGTAGAAGAGATGACACTCGGG -3′
IGF2BP3	Forward 5′- AGACACACTGAATCACCTGAAGT -3′<br>Reverse 5′- AGGGCGACACTGCTTTTTCTT -3′
YTHDF1	Forward 5′- GGGGACAAGTGGGTCTCAAG -3′<br>Reverse 5′- AGGGTGTCGCTGTGAAAGC -3′
YTHDF2	Forward 5′- GTTGGTAGCGGGTCCATTACT -3′<br>Reverse 5′- GGTCTTCAGTTTAGGTTGCTGT -3′
YTHDF3	Forward 5′- GGTGTATTTAGTCAACCTGGGG -3′

	Reverse 5′- AAGAGAACTAGGTGGATAGCCAT -3′
METTL3	Forward 5′- TTGTCTCCAACCTTCCGTAGT -3′<br>Reverse 5′- CCAGATCAGAGAGGTGGTGTAG-3′
METTL16	Forward 5′- CTCTGACGTGTACTCTCCTAAGG -3′<br>Reverse 5′- TACCAGCCATTCAAGGTTGCT -3′
WTAP	Forward 5′- CTTCCCAAGAAGGTTCGATTGA -3′<br>Reverse 5′- TCAGACTCTCTTAGGCCAGTTAC -3′
VIRMA	Forward 5′- CCTTCCTGGGCTAGTGCAAA -3′<br>Reverse 5′- TTTTGTTTCTTTTGGTACAGCCAT -3′
ZC3H13	Forward 5′- TCTGATAGCACATCCCGAAGA -3′<br>Reverse 5′- CAGCCAGTTACGGCACTGT -3′
RBM15	Forward 5′- ACGACCCGCAACAATGAAG -3′<br>Reverse 5′- GGAAGTCGAGTCCTCACCAC -3′
RBM15B	Forward 5′- ACCTGGACCACAGCGTATCT -3′<br>Reverse 5′- GGGTTGCGACCAATCACTC -3′
RBMX	Forward 5′- CTTCAGGACCAGTTCGCAGTA -3′<br>Reverse 5′- TCACGACCACTTGAGTAGAGAT -3′
YTHDC1	Forward 5′- CTTCTGATGAGCAAGGGAACAA -3′<br>Reverse 5′- GGCCTCACTTCGAGTGTCATAA -3′
YTHDC2	Forward 5′- CAAAACATGCTGTTAGGAGCCT -3′<br>Reverse 5′- CCACTTGTCTTGCTCATTTCCC -3′
HNRNPC	Forward 5′- TCCTCCTCCTATTGCTCGGG -3′<br>Reverse 5′- AGCCACTTTTGCCCCTTCG -3′
FMR1	Forward 5′- ACTTACGGCAAATGTGTGCCA -3′<br>Reverse 5′- GCAGACTCCGAAAGTGCATGT -3′
LRPPRC	Forward 5′- CGGAGGACTACTGAGCCCA -3′<br>Reverse 5′- AGCGGCAGGTATCATTAAAAACT -3′
HNRNPA2B1	Forward 5′- TGGAGGTAGCCCCGGTTATG -3′<br>Reverse 5′- GGACCGTAGTTAGAAGGTTGCT -3′
ALKBH5	Forward 5′- ATGCACCCCGGTTGGAAAC -3′<br>Reverse 5′- GACTTGCGCCAGTAGTTCTCA -3′

Table S2. Primary antibodies

Primary antibodies	Manufacturer, Catalogue number
Rabbit anti-m^{6}A	Synaptic Systems, 202003
Rabbit anti-LC3- I/II	Abcam, ab192890
Mouse anti-GFP	Abcam, ab1218
Rabbit anti-RFP	Abcam, ab185921
Rabbit anti-SQSTM1	Abcam, ab109012
Rabbit anti-ACTB	Abcam, ab8227
Rabbit anti-FTO	Abcam, ab126605
Rabbit anti-GAPDH	Abcam, ab9485
Rabbit anti-Ki67	Abcam, ab15580
Rabbit anti-SIK2	Abcam, ab245211
Rabbit anti- IGF2BP2	Abcam, ab128175
Rabbit anti-ATG5	Cell Signaling Technology, 9980S
Rabbit anti-ATG7	Cell Signaling Technology, 8558S
Rabbit anti-IGFBP2	Proteintech, 11065-3-AP
Rabbit anti-ALKBH5	Proteintech, 16837-1-AP
Rabbit anti-PIK3C2A	Proteintech, 22028-1-AP
Rabbit anti-N-cadherin	Proteintech, 66219-1-Ig
Rabbit anti-E-cadherin	Proteintech, 20874-1-AP
Rabbit anti-RNF34	ABclonal, A8517

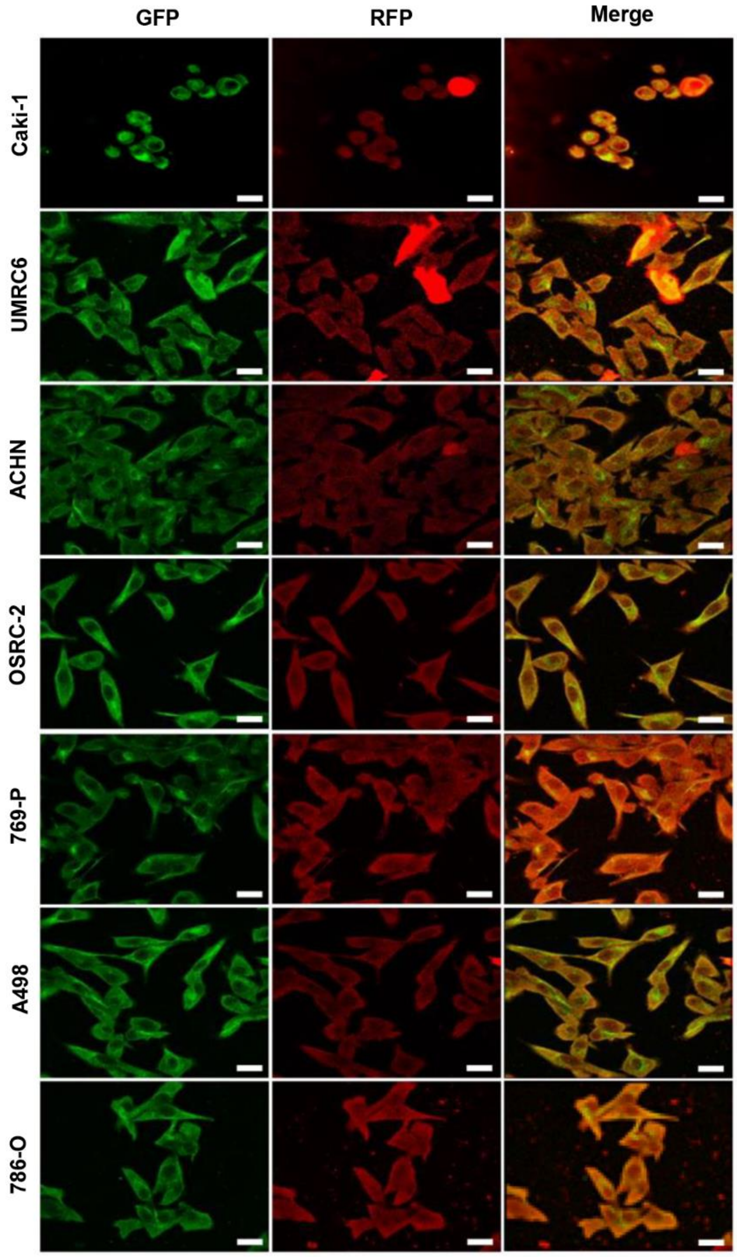

Figure S1. Immunofluorescence staining with mRFP-GFP-LC3 in ccRCC cell lines. Red puncta signify autolysosomes and yellow puncta signify autophagosomes. Scale bar, 10 μm.

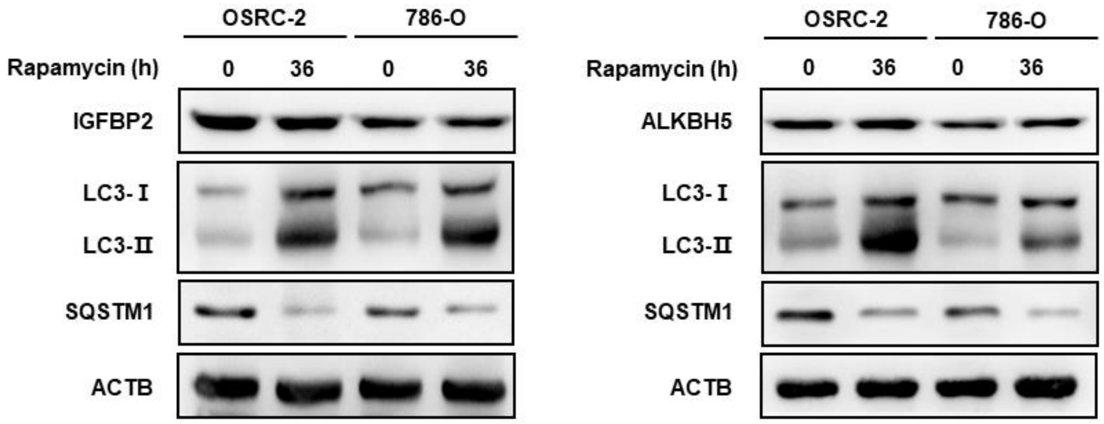

Figure S2. IGFBP2 and ALKBH5 expression in OSRC-2 and 786-O cells treated with rapamycin.

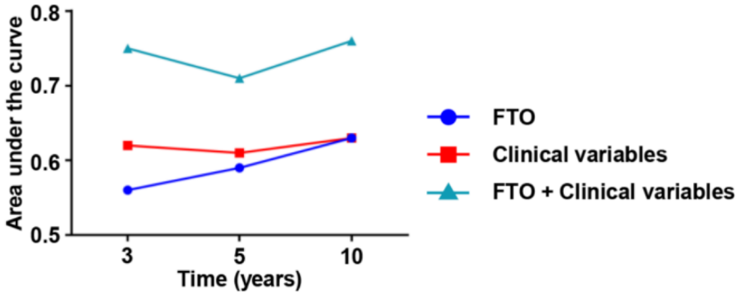

Figure S3. The time-dependent receiver operating characteristic (ROC) analysis for the FTO risk score, the clinical variables risk score, and the combined FTO and clinical variables risk scores in the ccRCC cohort.

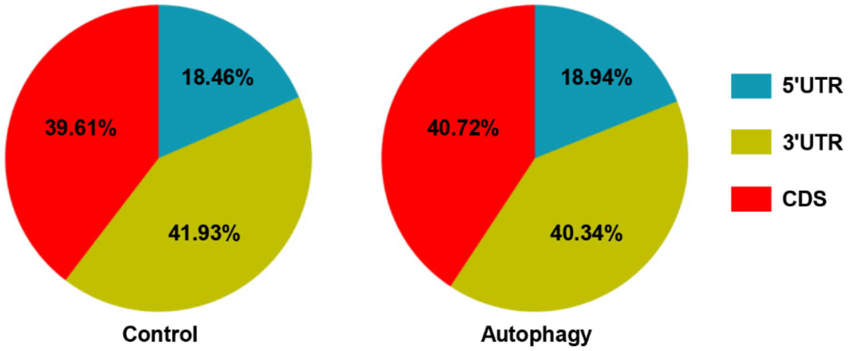

Figure S4. m^{6}A peak distribution in the 5′UTR, 3′UTR, and CDS across the entire set of mRNA transcripts.

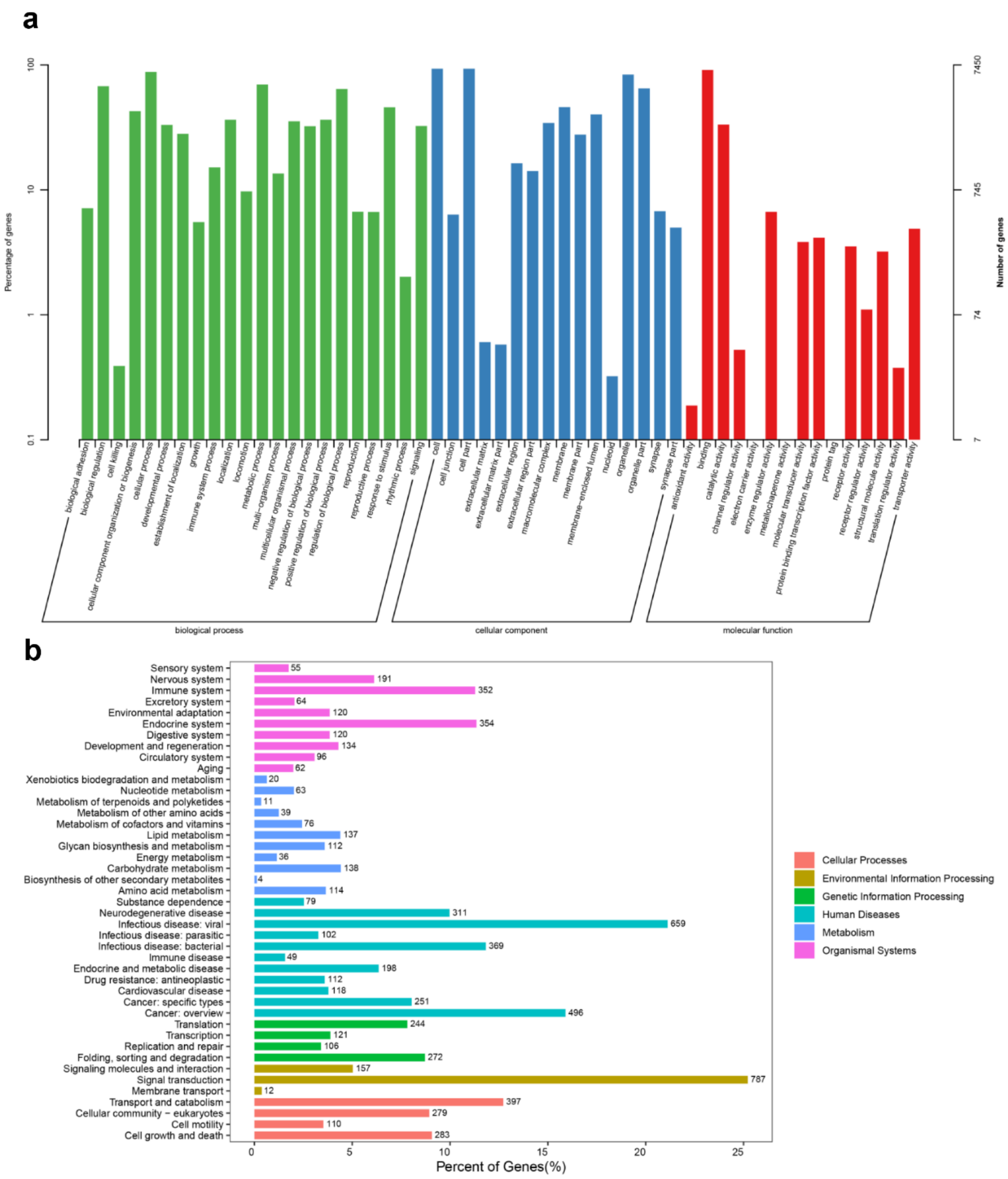

Figure S5. Gene Ontology pathway (a) and Kyoto Encyclopedia of Genes and Genomes pathway (b) analyses of differentially expressed genes in autophagy-induced OSRC-2 cells compared with normal OSRC-2 cells.

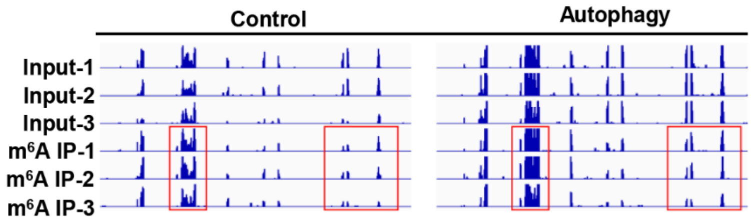

Figure S6. MeRIP-seq data revealed that m^{6}A abundance in the SIK2 mRNA was markedly increased during autophagy flux.

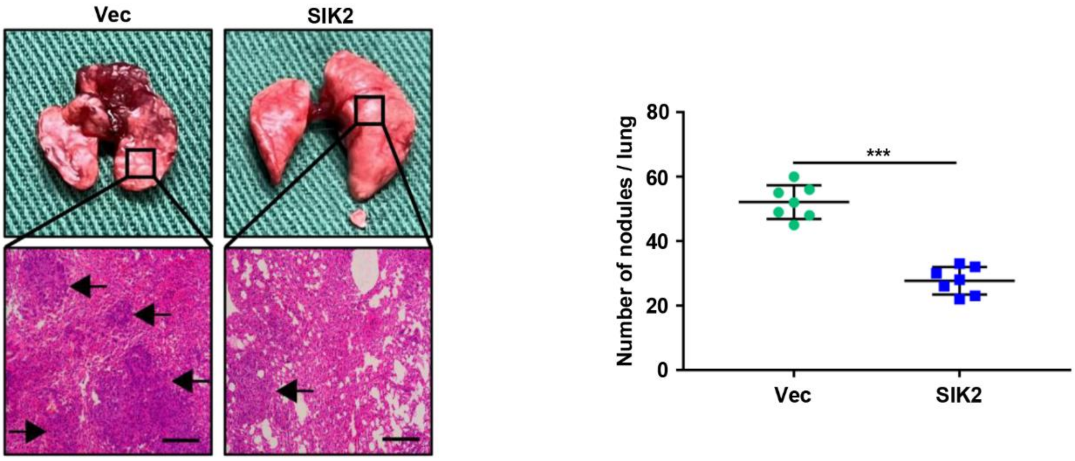

Figure S7. Representative images of metastatic lung tumors and hematoxylin and eosin (H&E) staining (left panel), and quantification of lung tumors (right panel).

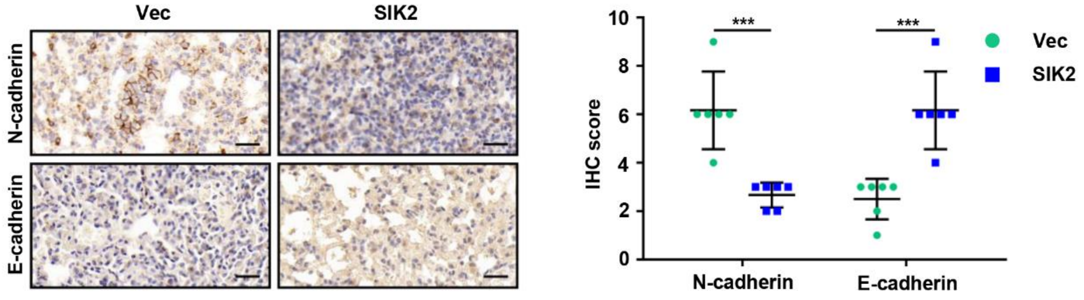

Figure S8. Representative IHC staining images of N-cadherin and E-cadherin (scale bar, 100μm, left panel) and quantification of IHC score (right panel). Error bars, SEM; ***, $P < 0.001$.
